# Umbilical Lesions: A Cluster of Known Unknowns and Unknown Unknowns

**DOI:** 10.7759/cureus.5309

**Published:** 2019-08-02

**Authors:** Aditi Das

**Affiliations:** 1 Pathology, Pandit Jawaharlal Nehru Memorial Government Medical College, Raipur, IND

**Keywords:** umbilicus, umbilical granuloma, umbilical papilloma, umbilical tumours, umbilical nodule, umbilical mass, umbilical hernia, exomphalos (omphalocele), sister joseph’s nodule, umbilical polyps

## Abstract

Umbilical lesions are rare, but it is important to cautiously inspect and investigate every umbilical nodule or growth to rule out the possibility of embryological remnant and associated congenital anomaly in infants and children and hidden malignancy in adults. Certain umbilical anomalies can be expected in association with certain syndromes (i.e., known unknowns), and at times can be identified during prenatal screening, while others are simply unforeseeable conditions that may arise unexpectedly (i.e., unknown unknowns). Umbilical lesions can be diagnosed on careful clinical and histopathological evaluation. Benign tumours are much more common than metastatic deposits. Certain lesions such as umbilical granuloma can be managed conservatively, while exomphalos and complicated umbilical hernia require urgent surgical intervention. This review article will help to elucidate the spectrum of umbilical lesions, with special emphasis on the importance of proper evaluation of often neglected, but clinically important entities.

## Introduction and background

The umbilicus is a relatively weak depression in the anterior abdominal wall, at the level of the highest points of the iliac crests, opposite the disk between L3-L4 vertebrae or L4 vertebra [1]. It appears when the embryonic plate folds, around the fourth week of intrauterine life [1]. The umbilicus is prone to herniation in the settings of high abdominal pressure, and it is prone to various developmental disorders given the presence of many embryological remnants [1-2]. In young children and adults, umbilical lesions demand meticulous investigation, as such lesions may reflect serious hidden underlying disorder or malignancy. While some require emergent intervention, others can be managed conservatively. Detecting such lesions at the earliest is crucial for preventing complications.

## Review

Congenital lesions

Umbilicus can be occasionally absent in certain congenital conditions like cloacal exstrophy, severe epispadias, and omphalocele [1]. Rarely, low umbilicus may be observed in Robinow syndrome, renal agenesis-dysplasia, fetal growth retardation, single umbilical artery, hydrops fetalis, anencephaly, monozygous twinning, and achondroplasia [1,3]. In infantile umbilical hernia, the intestine usually protrudes into the umbilical ring due to the persistence of communication between the fetal peritoneal cavity and extra-embryonic mesoderm caused by insufficient development of the umbilical ring. It is often associated with Down syndrome, prematurity, and mucopolysaccharidosis [1-2]. Most cases spontaneously resolve during early childhood, so the follow-up observation and parental reassurance are recommended. However, if the hernia is more than 1.5 cm, surgical repair is needed at age four to six years [1]. In exomphalos (omphalocele), abdominal contents bulge outside, and instead of skin, it is covered by a membrane composed of Wharton’s jelly, peritoneum, and amnion. It is associated with umbilical ring defect (of 5 to 15 cm) and is usually diagnosed prenatally by ultrasound. The visceral surface needs to be covered with saline-soaked gauzes, and prompt surgical repair should be attempted within hours after birth. If the sac ruptures, it requires emergent surgical intervention [4].

Vitelline duct’s remnants/anomalies and urachal remnants are summarized in Table 1 and Table 2, respectively.

**Table 1:** Vitelline duct’s remnants/anomalies

Anomaly	Cause	Feature
Vitelline (umbilical) fistula	Failure of normal obliteration of patent vitelline duct; directly communicates ileal cavity with exterior umbilical opening.	Ileal (fecal) content discharges through umbilicus; needs to be treated surgically.
Meckel’s diverticulum	Arises from ileum and is caused when proximal portion of vitelline duct, fails to obliterate.	Occurs in approximately 2% of the population [1,5] and often misdiagnosed as appendicitis.
Vitelline sinus	Distal (outer) portion of vitelline duct fails to obliterate.	A small blind opening at umbilicus, results in mucous discharge [1-2,5].
Vitelline cyst	Middle portion of vitelline duct fails to obliterate.	Cyst developed at the umbilicus may rupture or may get infected.
Vitelline band	Fibrous band connecting intestine with umbilicus, caused by obliteration of vitelline duct.	Cause intestinal volvulus, strangulation and obstruction [5].
Mucosal remnants	Includes ectopic mucosa, gastric or pancreatic in origin, and rarely colonic mucosa [6].	Associated with umbilical polyp or umbilical cyst.

**Table 2:** Urachal remnants/anomalies *If nature of discharge is bilious or fecal, prompt work-up should be performed to exclude persistent omphalomesenteric duct [7].

Anomaly	Cause	Feature
Urachal sinus	Incomplete obliteration of urachus.	Periumbilical discharge*
Patent urachus	Urachus, an embryonic duct extending from bladder to umbilicus, remains patent [7].	Periumbilical discharge or umbilical granulomas or polyp; intermittently leaks urine.
Urachal cyst	Middle portion of urachus fails to obliterate.	Periumbilical mass in childhood; may get infected which can be treated by drainage [2,7].
Urachal fistula	Urachus fails to obliterate completely.	Discharge of urine at umbilicus; may be treated by cauterization. Surgical ligation and excision done, if it persists.
Urachal diverticulum	Proximal part of urachus fails to obliterate.	Usually asymptomatic.

Umbilical granuloma

Umbilical granulation tissue is often seen in newborns after cord separation , and when in excess, it may turn into simple pyogenic granulomas-the most common neonatal umbilical anomaly [7]. The condition may also be caused by delayed and irregular separation of the cord stump. Umbilical granulomas are solid, soft, velvety red in appearance. They generally develop within the first few weeks of life and can present as a pedunculated mass with serosanguineous discharge, approximately 1 mm to 10 mm in diameter. It is usually treated with 75% silver nitrate or can be surgically excised. Failure to respond to silver nitrate application differentiates these granulomas from granulomas secondary to patent urachus, polyps, and other embryological remnants [7].

Umbilical polyps 

They are rare, appear as a bright red nodule, may be associated with Meckel’s diverticulum or other omphalomesenteric duct anomalies, and can be a clue to ensuing life-threatening intestinal obstruction. They do not respond to silver nitrate application. Histologically, umbilical polyps are lined by intestinal mucosa [8].

Other miscellaneous umbilical lesions include epithelial inclusion cyst, endometriosis, and, very rarely, condyloma accuminata [9-10]. Endometriosis can involve umbilical skin and can mimic a sweat gland tumour and/ or metastatic adenocarcinoma. Histopathological analysis is necessary which confirms the presence of both endometrial glands and stroma in endometriosis [11].

Umbilical tumours

Umbilical tumours are encountered only in adults. They can be divided into benign and malignant. Benign tumours are most common, constituting 57% of all umbilical tumours, followed by metastatic deposits [9-10]. Primary benign umbilical tumours are melanocytic tumors, fibroepithelial papillomas, seborrhoeic keratoses, dermatofibromas, neurofibromas, juvenile hemangiomas, keloid, granular cell myoblastoma, desmoids, and lipoma [9-10]. Primary malignant umbilical tumours are very rare, constituting only 17% of cases and the spectrum includes melanoma, basal cell carcinoma and adenocarcinoma, while metastatic umbilical carcinomas constitute 83%, and, in most cases, have their primary in the gastrointestinal tract [9-11].

Umbilical Papillomas

Umbilical papilloma is a rare, primary benign tumour and has only been reported in adults. However, umbilical papillomas justify separate mention here because the forerunner of the adult umbilical papilloma is presumed to be a neglected umbilical granuloma. If an umbilical granuloma develops in a neonate and is allowed to persist for a long time, the granuloma may eventually epithelialize and form papilloma later [12]. Congenital umbilical papillomas are presumed to be due to mother-fetal human papillomavirus transmission. They are asymptomatic, slow- growing, and black, warty in appearance. They are usually not associated with any discharge or ulceration. Clinical and histopathological evaluation is required for diagnosis. On histopathology, they display features of benign verruciform papillomas characterized by the presence of central fibromuscular stalk with loose connective tissue surrounded by benign squamous epithelium, with no significant cytologic atypia.

Sister Mary Joseph’s Nodule

The term “Sister Mary Joseph’s nodule” is used for metastatic umbilical tumors presenting as irregular, nontender, firm, bluish-violet or brownish-red nodules [9]. They can be fissured or ulcerated and associated with bloody, mucinous, serous, or purulent discharge. A Sister Mary Joseph’s nodule is typically less than 5 cm in diameter; however, it may enlarge to form a protruding tumour. Hence, histopathological evaluation is necessary for confirmation, which generally reveals metastatic adenocarcinoma. Most often metastasis occurs from gastric adenocarcinoma in men and from ovarian cancer in women; however, metastasis from sarcomas, melanomas and mesotheliomas have also been reported [9,13]. It signifies advanced metastasizing malignancy associated with poor prognosis and, therefore, requires urgent attention [9].

## Conclusions

The knowledge of the age of presentation and characteristic clinical features of umbilical lesions helps in establishing the diagnosis. Fine needle aspiration cytology may also be attempted, however in doubtful cases, histopathology remains the gold standard. Histopathological evaluation is necessary because it carries a diagnostic and prognostic significance in umbilical lesions. Any umbilical growth or nodule should be evaluated diligently, especially in adults, as it can be the only clue to underlying hidden malignancy.
